# Relationships between Adipose Tissue and Psoriasis, with or without Arthritis

**DOI:** 10.3389/fimmu.2014.00368

**Published:** 2014-08-12

**Authors:** Éric Toussirot, François Aubin, Gilles Dumoulin

**Affiliations:** ^1^Clinical Investigation Center for Biotherapy INSERM CIC-1431, University Hospital of Besançon, Besançon, France; ^2^Department of Rheumatology, University Hospital of Besançon, Besançon, France; ^3^Department of Therapeutics, University of Franche Comté, Besançon, France; ^4^UPRES EA 4266 “Pathogens and Inflammation”, University of Franche Comté, Besançon, France; ^5^LabEX LipSTIC, ANR-11-LABX-0021, Besançon, France; ^6^Department of Dermatology, University Hospital of Besançon, Besançon, France; ^7^University of Franche-Comté, Besançon, France; ^8^Endocrine and Metabolic Biochemistry, University Hospital of Besançon, Besançon, France; ^9^UPRES EA 3920 “Cardiovascular Pathophysiology and Prevention”, University of Franche Comté, Besançon, France

**Keywords:** obesity, psoriasis, psoriatic arthritis, cardiovascular risk, adipokines

## Abstract

Psoriasis (Pso) is a common chronic cutaneous inflammatory disease involving the skin that is associated with serious comorbidities. Comorbidities in Pso include psoriatic arthritis (PsA), reduced quality of life, malignancy, depression, but also a constellation of associated conditions that enhance the cardiovascular (CV) risk. Indeed, obesity is common in patients with Pso or PsA and is considered to be a risk factor for the onset of these diseases. Patients with Pso and PsA share common obesity-related complications such as metabolic syndrome (MetS), dyslipidemia, diabetes or insulin resistance, and CV diseases. Chronic inflammation in Pso and PsA partially explains the development of atherosclerosis and CV diseases. In parallel, body composition is disturbed in patients with Pso or PsA, as suggested by anthropometric measurements, while an excess of abdominal adiposity is observed in PsA, enhancing the risk of MetS and CV diseases. Adipokines may link the adipose tissue to the obesity-related complications of Pso and PsA. Indeed, altered circulating levels of the adipokines leptin, adiponectin, visfatine, and resistin have been found in patients with Pso or PsA. In addition, an excess of adipose tissue may compromise the therapeutic response to traditional drugs or biological agents in Pso and PsA. This paper reviews the comorbidities that contribute to enhanced CV risk, the body composition results, and the potential role of adipokines in systemic inflammation and energetic balance in Pso and PsA.

## Introduction

Psoriasis (Pso) is a chronic cutaneous immune-mediated disease that affects around 1–3% of the adult population in Europe and North America. Its pathophysiology involves genetic and environmental factors together with immune disturbances. Pso is a papulosquamous disease with variable morphology, distribution, severity, and course. Cutaneous lesions are characterized by very well circumscribed scaling papules and plaques that are typically distributed symmetrically on the knees, elbows, scalp, genitals, and trunk (1). Different clinical forms of Pso are described including plaque Pso, guttate Pso, nail Pso, Pso inversa, localized and generalized pustular forms, and erythrodermic Pso. There is substantial evidence demonstrating that Pso is associated with various comorbidities, such as depression, cancer, obesity, and cardiovascular (CV) diseases (2). Indeed, an increased risk of coronary, cerebrovascular, and peripheral vascular diseases has been reported in patients with severe Pso (2).

Another common comorbidity in Pso is psoriatic arthritis (PsA), an inflammatory arthritic condition (3). The prevalence of PsA is estimated at between 6 and 40% in the psoriatic population. PsA is classified in the spondyloarthritis group and is commonly characterized by synovitis, enthesitis, dactylitis, and spondylitis. Typically, the skin disease occurs before the onset of arthritis in more than 80% of patients. Nail dystrophy, Pso of the scalp, and/or intergluteal/perianal Pso have been identified as clinical predictors for the development of arthritis (4). PsA is currently diagnosed using the ClASsification for Psoriatic ARthritis (CASPAR) criteria, with the requirement of established inflammatory articular disease plus at least three points from the following: current Pso (assigned a score of 2; all other features assigned a score of 1), a history of Pso, a family history of Pso, dactylitis, juxta-articular new bone formation, negative rheumatoid factor, and nail dystrophy (5). These criteria are highly specific and sensitive. The clinical presentation of PsA can manifest as peripheral arthritis, asymmetrical oligoarthritis, arthritis mutilans, or ankylosing spondylitis. The severity of PsA is variable, ranging from mild to severely debilitating disease with joint damage and functional impairment (3). PsA may also manifest as extra-articular disease with anterior uveitis and various comorbidities including depression, obesity, metabolic syndrome (MetS), and CV disease (6).

The pathogenesis of Pso and PsA includes common pathways with the implication of Th1, Th17, and Th22-mediated inflammation, inducing the production of pro-inflammatory cytokines such as Il-17 and TNFα (1, 3). Therapeutic management targeting TNFα and IL-23 (TNFα inhibitors and ustekinumab, an anti-IL12/23 p40 monoclonal antibody, respectively) has been proven to be highly effective in both Pso and PsA (7). In parallel to these common pathophysiological mechanisms, Pso and PsA also share common predisposing factors and/or comorbidities, especially obesity and related metabolic complications, as well as a higher risk of CV disease (6, 8). Obesity is characterized by chronic low grade inflammation and is considered to promote a low grade inflammatory state. Adipose tissue is in fact an active organ responsible for the production of a wide range of proteins or adipokines that have diverse physiological functions, including involvement in chronic inflammation (9).

In this review, we aim to discuss the role and influence of adipose tissue on Pso and PsA, adipokines, and body composition changes in these patient populations. The pattern of CV comorbidity in Pso and PsA as well as the influence of obesity on therapeutic response are also described.

## Cardiovascular Risk Factors and Comorbidities in Psoriasis and Psoriatic Arthritis

There is a constellation of comorbid conditions that are experienced by patients with Pso, with a predominance of CV and metabolic diseases (2). A modest increase in lymphoma including Hodgkin’s and cutaneous T cell lymphoma have been reported in patients with Pso in a large controlled cohort study (10). Solid organ malignancies (liver, pancreas, lung, breast, kidney) also occur in Pso with an increased risk. The use of psoralen ultraviolet and cyclosporin have been proposed as predisposing factors for the development of these malignancies (11). Impaired quality of life and depression are also frequent in patients with Pso (2). However, the main comorbidity in patients with Pso concerns CV involvement. In fact, there are a number of epidemiological studies in Pso demonstrating a high prevalence of CV diseases, including myocardial infarction, cerebrovascular diseases, and peripheral vascular diseases (12–14). Traditional CV risk factors are also commonly observed with a high frequency in patients with Pso, contributing to the CV burden. Among these factors are MetS, diabetes mellitus, insulin resistance, hypertension, dyslipidemia, and smoking (15–17). The MetS is defined by the presence of three or more features among the following: abdominal obesity, insulin resistance, decreased HDL cholesterol, hypertriglyceridemia, and hypertension (18). There are a substantial number of studies demonstrating that patients with Pso have these components of the MetS, largely contributing to their CV risk (2, 15). Hypertension is more often observed in patients with Pso as compared to a control population, although this association remains controversial (2). Pso is associated with dyslipidemia with higher concentrations of triglycerides, total cholesterol, and LDL cholesterol (16). Increased prevalence of type 2 diabetes is observed in Pso, with a significant correlation between the duration of Pso and glucose intolerance (17). CV diseases and related mortality have been reported in patients with severe Pso both in cohort and population-based studies. Overall, these studies show an increased risk of various CV diseases, such as myocardial infarction, stroke, and peripheral vascular disease (2). A recent systematic literature review of CV morbidity and mortality in Pso identified 33 observational studies in patients with Pso and PsA and found an increased risk of myocardial infarction in Pso with an odds ratio (OR) of 1.25 (95% CI: 1.03–1.52), compared to the general population (19). This risk was more elevated in patients with severe skin disease and early onset. An association was also found between Pso and coronary artery disease, with an OR ranging from 1.19 to 1.80 according to the design of the studies. On the contrary, the increase in the risk of stroke was less pronounced, with a slightly higher risk (OR: 1.14, 95% CI: 1.08–1.99). In this systematic review, peripheral artery disease was found to be associated with Pso, but the limited number of studies precluded estimation of the OR.

Psoriatic arthritis is also associated with various comorbidities, including depression, obesity, MetS, hypertension, and premature CV disease (6, 20, 21). The association between PsA, CV diseases, and comorbidities is complex and not well understood. In fact, in terms of CV risk, several studies have demonstrated a higher risk in patients with Pso but data on CV disease more specifically in PsA are limited. Theoretically, the coexistence of skin disease with arthritis in patients with PsA may be responsible for a greater inflammatory load and consequently, these patients may be more prone to developing CV disease compared to patients with only skin involvement. A systematic literature search analyzed CV comorbidities and CV risk factors in patients with PsA (22). Twenty-eight articles were selected in this analysis, which revealed mixed results for CV mortality. Among three observational studies, two showed a higher standardized mortality rate (1.6 and 1.4, respectively) while the third did not observe an increase in mortality rate. The same findings were observed with population-based studies. On the contrary, results for CV disease and risk factors were more consistent, indicating increased CV morbidity in PsA, including ischemic heart disease, congestive heart failure, cerebrovascular, and peripheral vascular disease. This review indicated that traditional CV risk factors were commonly observed in patients with PsA, with an increased prevalence of hypertension, obesity, diabetes mellitus, and dyslipidemia. Indeed, hypertension was found in 30% of all PsA patients. However, this review analyzed only data in patients with PsA without parallel assessment of Pso patients without arthritis and no OR for the increased CV risk was available. Conversely, a study by Husted et al. compared CV morbidity in PsA vs. Pso without arthritis (23). This study was performed in Canada from a cohort of Pso without arthritis cohort and a PsA cohort. The results showed that the prevalence of hypertension, obesity, hyperlipidemia, type 2 diabetes mellitus, and at least one CV event was higher in patients with PsA compared to Pso, with unadjusted OR ranging from 1.54 to 2.59. The main comorbidity that remained significantly elevated in PsA after adjustment for confounding factors was hypertension (adjusted OR: 2.17, 95% CI: 1.22–3.83). Similarly, the literature review by Horreau et al. compared CV morbidity and mortality in patients with PsA and Pso compared to controls (19). An increased risk of myocardial infarction was noted in both groups, with PsA patients at higher risk (OR 1.57, 95% CI: 1.08–2.27 in PsA compared to 1.25, 95%, CI: 1.03–1.52 in Pso). An increased risk of coronary heart disease was again observed in Pso and PsA, but the limited number of studies did not allow quantification of this risk. In addition, this analysis did not observe an increased risk of CV mortality in patients with Pso or PsA.

## Obesity in Psoriasis and Psoriatic Arthritis

A strong association between increased body weight, adiposity, and Pso has recently emerged (8). Indeed, several studies have consistently underlined a significant link between body mass index (BMI) and Pso. A case–control study performed in Italy found an association between BMI and Pso of recent onset, with an OR of 1.6 (95% CI: 1.1–2.1) and 1.9 (95% CI: 1.2–2.8) for overweight (BMI ranging from 26 to 29 kg/m^2^) and obese patients (BMI ≥30 kg/m^2^), respectively (24). Similarly, the prevalence of obesity in patients with Pso was found to be twice as high as that in the general population in the Utah Pso initiative study (25). The relationship between BMI, weight change, and waist circumference was examined in women included in the Nurse’s Health study in a prospective longitudinal study over a 14 year period. This study demonstrated a graded positive association between BMI evaluated at multiple time points and the risk of incident Pso. Similarly, measurements of visceral adiposity (waist circumference, waist-to-hip ratio) and weight gain were identified as strong predictors of Pso development (26). This study provides strong evidences that increased adiposity is a risk factor for incident Pso. Conversely, certain studies reported that BMI increased in patients after Pso diagnosis, suggesting that obesity is secondary to Pso (25). Whether adipose tissue is a risk factor for Pso development or a secondary feature after its onset still remains debated. However, cross-sectional studies have indicated that obesity is more prevalent in patients with severe Pso than in patients with mild disease, reinforcing the link between fat mass and Pso (17, 27).

In parallel to its influence on Pso, obesity also plays a role in PsA (6). Indeed, an increased prevalence of obesity has been reported in PsA as compared to the general population. A case–control study in patients with Pso found that BMI at age 18 years, and not current BMI, was associated with the development of PsA (28). A cohort study using an electronic database of medical records and representative of the general population in the United Kingdom analyzed the impact of the first BMI measured after Pso diagnosis on incident PsA (29). This study found that PsA incidence rates increased in tandem with increasing BMI: compared with Pso patients with BMI <25 kg/m^2^, the relative risk (RR) for developing PsA was 1.09 (95% CI: 0.93–1.28) for BMI from 25 to 29.9 kg/m^2^, 1.22 (95% CI: 1.02–1.47) for BMI from 30 to 34.4 kg/m^2^, and 1.48 (95% CI 1.2–1.81) for BMI ≥35 kg/m^2^. These results were observed both in subjects with Pso and in the general population, independently of age, sex, trauma, smoking status, and alcohol intake (29). Another study examined the risk of incident PsA in US women participating in the longitudinal Nurse’s Health II study cohort, over a 14 year period. The results showed a strong relationship between BMI and increased incident risk of PsA: compared with BMI <25 kg/m^2^, the RR was 1.83 (95% CI: 1.15–2.89) for BMI from 25 to 29.9 kg/m^2^, 3.12 (95% CI: 1.90–5.11) for BMI from 30 to 34.9 kg/m^2^, and 6.64 (95% CI: 4.11–10.16) for BMI >35 kg/m^2^. Moreover, there was a graded positive association between weight changes from age 18 years, measures of central obesity (as evaluated by waist circumference and hip to waist ratio) and the risk of incident PsA (30). These two studies, thus, provide evidence linking obesity to the risk of incident PsA. In addition, the prevalence of obesity was higher in patients with PsA compared to patients with Pso in the study by Husted et al. (unadjusted OR: 1.58, 95% CI: 1.19–2.09) (23).

## Body Composition in Psoriasis and Psoriatic Arthritis

Data on body composition in patients with Pso or PsA are sparse. Anthropometric measurements such as waist circumference or waist-to-hip ratio reflect central or abdominal adiposity and have been proposed as substantial indicators of health risk. These indices have been recorded in patients with Pso or PsA. In the study by Setty et al., waist circumference and waist-to-hip ratio were associated with the risk of incident Pso (26). Similarly, these same measures of visceral adiposity were reported to be associated with the development of PsA (30). Children with Pso had excess adiposity and increased central adiposity as evaluated by waist circumference and waist-to-height ratio, regardless of Pso severity (31). Fat mass and its distribution may be more specifically evaluated by dual-energy X ray absorptiometry (DXA), the reference method for body composition measurements. However, a limited number of studies have evaluated body composition in patients with Pso using DXA. In a series of Brazilian patients with Pso, total body fat was similar between psoriatic patients and healthy controls (32). In the same study, patients with PsA had more evidence of adiposity with a significantly higher body fat percentage than Pso and healthy controls. In these patients, the excess of fat tissue was located in the android region.

## Adipokines in psoriasis and psoriatic arthritis

Adipose tissue is mainly composed of adipocytes, with, in parallel, a stromal–vascular fraction, which includes macrophages. Adipocytes are able to secrete a wide range of proteins called adipokines, which are involved in different physiologic processes. Besides these functions, adipokines also interact with immune cells, therefore, contributing to the inflammatory network (9). For instance, the adipokines, such as leptin, visfatin, and resistin, display pro-inflammatory activities. On the contrary, adiponectin is believed to have anti-inflammatory effects, especially, its low molecular weight isoform. Adipose tissue is also a substantial source of pro-inflammatory cytokines such as IL-6, TNFα, and IL-8. In parallel to their relationships with the immune system, adiponectin, visfatin, and resistin have metabolic functions and play a role in insulin sensitivity (33). Moreover, clinical studies suggest a regulatory and anti-inflammatory role for adiponectin in atherosclerosis. Thus, by affecting vascular function, immune regulation, and glucose metabolism, adipokines are considered as key players in the pathogenesis of obesity-related complications such as MetS and CV morbidity. The role and influence of fat tissue and adipokines is increasingly studied in chronic immune-mediated diseases, such as chronic inflammatory skin or rheumatic diseases (9, 34). Therefore, the assessment of fat distribution and adipokine production is a relevant issue in conditions associated with an excess of fat, such as Pso and PsA.

In patients with Pso, circulating adiponectin (total adiponectin) levels have been shown to be decreased as compared to healthy controls (35, 36). A negative correlation between adiponectin and extent of the skin disease evaluated by psoriasis area severity index (PASI) or pro-inflammatory cytokines, such as IL-6 and TNFα was found in these studies (35, 36). On the contrary, leptin levels were increased in both male and female patients with Pso (37). In an immunohistochemistry study, the cutaneous expression of leptin and leptin receptor correlated with severity of Pso and disease duration (38). Similarly, resistin concentration was increased in patients with Pso and correlated with disease severity (39). In a gene expression study performed in peripheral blood mononuclear cells from patients with Pso, the visfatin gene was found strongly up-regulated (40). Retinol-binding protein-4 (RBP-4) is mainly produced by visceral adipose tissue and plays a major role in insulin resistance. It has previously been demonstrated that serum RBP-4 positively correlates with several CV risk factors, such as BMI, waist-to-hip ratio, serum triglycerides, and systolic blood pressure and may thus play a role in CV diseases in obese subjects (41). In patients with Pso, surprisingly, decreased serum RBP-4 concentrations have been found compared to healthy controls (42). Omentin is a protein produced by stromal–vascular cells of visceral adipose tissue and it increases insulin sensitivity by stimulating insulin-mediated glucose uptake in human adipocytes. Since serum levels of omentin inversely correlate with fat mass, omentin is regarded as a positive marker that counteracts the obese state. Omentin serum levels were found to be decreased in patients with Pso and negatively correlated with BMI and waist circumference, as expected (43). A study conducted in Canada compared serum adipokine levels between patients with PsA and patients with Pso without arthritis. This study found that the prevalence of MetS was higher in the PsA group (36.5 vs. 27.1%) without reaching statistical significance (*p* = 0.056). Serum adipokine levels were higher in patients with PsA compared to Pso, especially, adiponectin and leptin in women (44). In another study, patients with PsA had higher serum levels of leptin and omentin but decreased adiponectin levels compared to healthy controls (45).

## Influence of Biological Agents on Adiposity and Influence of Adiposity on the Therapeutic Response in Psoriasis and Psoriatic Arthritis

We previously reported that anti-TNFα therapy given in patients with rheumatoid arthritis (RA) or spondyloarthritis is associated with a significant increase in body weight, BMI, fat mass, and especially fat mass in the android region (46). Our results also suggested that this fat mass distributed to the intra-visceral region. This fat redistribution raises questions about its influence on the CV profile of patients receiving this drug class. Body weight and body composition changes have also been assessed in patients with Pso or PsA while receiving anti-TNFα treatment. In a longitudinal study of 24 weeks duration, 40 patients with Pso or PsA were treated by infliximab or etanercept, 2 TNFα inhibitors. Compared to baseline, the results showed a significant gain in body weight in both groups (+2.6 and +2.1% in Pso and PsA, respectively), an increase in fat mass in the Pso group (+8.5%), and in both fat mass (+8.9%) and lean mass (+2.9%) in the PsA group (47). However, in this study, fat distribution (android or visceral region) was not analyzed. In a retrospective analysis of patients with chronic plaque Pso, a body weight increment (+1.5 to +2.5 kg) was observed in patients receiving anti-TNFα agents for 6 months, while no change was observed for those under methotrexate. In parallel, BMI also increased (+ 0.5 to +0.8) and about a quarter of patients had a 4–10 kg weight gain during this study (48). In addition, it has been suggested that weight gain may be higher with etanercept than with infliximab (49). On the contrary of TNFα inhibitors, ustekinumab, was not associated with weight or BMI change in patients with Pso (50).

On the other hand, obesity may influence the therapeutic approach and clinical response to systemic treatment in Pso or PsA, especially, the response to anti-TNFα treatment. Indeed, adipose tissue may alter the volume of drug distribution, and thus limit drug efficacy (20). Since biological drugs are usually administered at a fixed dose, a weight-adjusted dose is probably necessary for overweight/obese patients. In this sense, beneficial effects of weight loss on skin disease have been suggested in case reports of Pso clearing with reduction of obesity (51). A recent review analyzed the available literature on the biological treatment response according to body weight in patients with Pso. The conclusion was that optimal treatment response with fixed dose biological agents was less frequent in patients with increasing weight, especially above 100 kg (52). The same results were found for patients with PsA. In a prospective follow-up of 135 obese patients with PsA and 135 normal weight PsA controls, the presence of abdominal obesity was associated with an increased risk of not achieving the minimal disease activity (MDA) (53). The same authors demonstrated that a low-calorie diet intervention in overweight or obese patients with PsA was associated with a higher rate of MDA achievement (54). Another study confirmed this relationship between fat mass and clinical response in PsA in a Canadian population of 557 patients with PsA. The authors reported a dose response association between obesity and the probability of having clinical response to treatment [disease modifying anti-rheumatic drugs (DMARD) and/or TNFα inhibitor], with the patients in the higher BMI category less likely to achieve MDA compared to those in the lowest BMI category after adjustment for confounding variables (55).

## Relationships between Obesity, Psoriasis, and Psoriatic Arthritis

**Figure 1:**
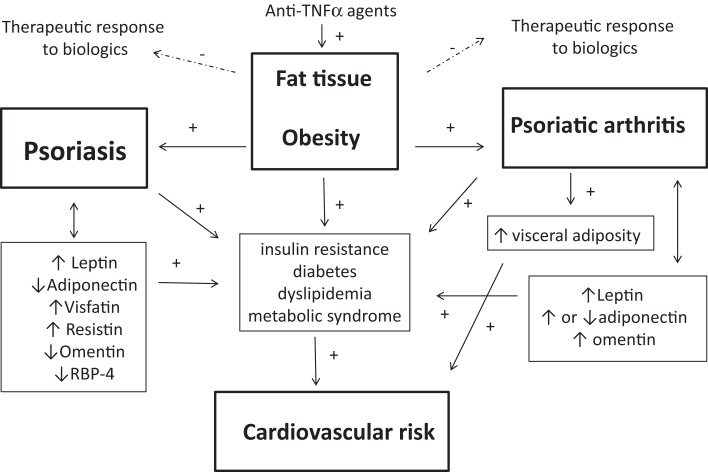
**Interrelationships between fat tissue, psoriasis (Pso), and psoriatic arthritis (PsA) are shown**. Obesity is a predisposing factor the development of both Pso and PsA. Pso and PsA are associated with obesity-related complications such as metabolic syndrome, dyslipidemia, diabetes, or insulin resistance, which all enhance the cardiovascular (CV) risk. Body composition is disturbed in Pso or PsA, especially in patients with PsA who have an excess of abdominal adiposity, contributing to increase the risk of metabolic syndrome and CV diseases. Adipokines may link the adipose tissue to the obesity-related complications of Pso and PsA: altered circulating levels of the adipokines leptin, adiponectin, visfatin, resistin omentin, and retinol-binding protein-4 (RBP-4) have been found in patients with Pso or PsA. Leptin has pro-inflammatory effects and may contribute to skin and joint inflammation. Adiponectin promotes insulin sensitivity and its reduced level in Pso may drive insulin resistance and may lead to impaired cardiac and vascular protective effects, thus contributing to the CV risk. In addition, the excess of adipose tissue in Pso and PsA may compromise the therapeutic response to biological agents in Pso and PsA. Finally, anti-TNFα agents have been associated with weight gain in patients with Pso or PsA.

There is compelling evidence suggesting that both Pso and PsA are associated with an excess of fat tissue (8, 20) (Figure 1). Moreover, obesity-related comorbidities accumulate in both conditions, especially, CV and metabolic comorbidities, leading to an increased CV burden. Pro-inflammatory cytokines, such as TNFα and IL-6, and the adipokines leptin, resistin, and visfatin produced by fat tissue are contributing and probably synergistic factors to a pro-inflammatory state in these conditions. Both Pso and PsA are associated with body composition changes, mainly in PsA. One unanswered question is the timing of adipose tissue changes: BMI and weight in early adulthood seem to predict Pso and PsA and conversely, patients with established disease (Pso or PsA) are characterized by excess fat mass. In other words, it remains to be clarified whether fat mass plays a role at the beginning (or before onset) of the disease, or whether the systemic inflammation in these chronic conditions may govern weight modification and fat redistribution.

The adipokines produced by adipose tissue in the context of Pso or PsA have a wide range of targets and consequences. An excess of fat mass results in the production of chemokines such as monocyte chemoattractant protein-1 (MCP-1), which contributes to increasing resident macrophages in fat tissue. This in turn may perpetuate the pro-inflammatory state (6). Adipose tissue may produce pro-angiogenic factors such as vascular endothelial growth factor, contributing to the skin and synovial pathogenesis in Pso and PsA, respectively (8). Adiponectin is believed to have anti-inflammatory effects (at least its low molecular weight fraction). Low serum levels of (total) adiponectin have been observed in Pso (but not in PsA) and a reduced level of adiponectin is associated with insulin resistance an impaired cardiac and vascular protective effects. These reduced adiponectin levels may thus contribute to the CV risk in Pso. According to cross-sectional studies and even after adjustment for sex and BMI, an excess of leptin characterized both Pso and PsA patients, contributing to the obesity-related inflammation. Leptin has also pro-angiogenic activities and is involved in the induction of endothelial dysfunction. Therefore, high leptin levels may accelerate atherosclerosis in patients with Pso and PsA. The high levels of visfatin and resistin also participate in the pro-inflammatory state and insulin resistance in Pso and PsA. More data are needed to fully understand the role of RBP-4 and omentin in Pso and PsA and their influence on CV comorbidities. In addition, there is no available data on the different adiponectin fractions in Pso or PsA. Collectively, these results strongly support the fact that obesity and fat tissue have the potential to initiate and drive many of the known inflammatory mechanisms underlying the pathogenesis of Pso and PsA and also their cardio-metabolic complications. Moreover, the importance of adipose tissue seems to be greater in PsA than in Pso and greater than in other inflammatory conditions. Indeed, a recent report showed that patients with PsA had the highest BMI compared to patients with Pso, RA, or the general population (56).

## Conclusion

The relationships between obesity, Pso, and PsA represent a challenging issue to be addressed. Obesity may determine both Pso and PsA development. Pso and PsA share a common serum adipokine profile, body composition changes, and cardio-metabolic complications, also with similar clinical outcomes such as the likelihood to achieve reduced disease activity during treatment with DMARDs and/or biologics. Obesity in both Pso and PsA is thus an important concern that must be adequately recognized and managed by clinicians. In addition, obesity-related complications are more prevalent in patients with PsA compared to Pso patients without arthritis. Thus, clinicians must carefully assess adipose tissue in patients with Pso and especially in those with concomitant arthritis, by evaluating weight, BMI, and the other clinical indices of visceral adiposity, in order to estimate the CV risk in these patients. Accordingly, weight loss should also be obtained in overweight/obese patients. This should contribute to reducing the CV risk, improving therapeutic response to DMARDs and/or biologics, improving mobility and thus physical activity, and ultimately should improve the long term prognosis of these patients.

## Conflict of Interest Statement

The authors declare that the research was conducted in the absence of any commercial or financial relationships that could be construed as a potential conflict of interest.
